# Multi-omics revealed that the postbiotic of hawthorn-probiotic alleviated constipation caused by loperamide in elderly mice

**DOI:** 10.3389/fnut.2025.1498004

**Published:** 2025-02-18

**Authors:** Yu Wei, Shuai Chen, Ying Ling, Wei Wang, Yali Huang

**Affiliations:** ^1^Guangzhou University of Chinese Medicine, Guangzhou, China; ^2^Basic Medical Science College, Guangzhou University of Chinese Medicine, Guangzhou, China; ^3^The First Clinical Medical School, Guangzhou University of Chinese Medicine, Guangzhou, China; ^4^Yunnan University of Chinese Medicine, Kunming, China

**Keywords:** postbiotic, hawthorn, probiotic, constipation, inflammation, intestinal microenvironment

## Abstract

**Background:**

Constipation is a prevalent and recurrent gastrointestinal disorder causing significant discomfort. However, current treatments often prove ineffective. Previous research indicates that the postbiotic derived from a combination of hawthorn and probiotics can alleviate constipation. This study aimed to investigate its mechanisms using loperamide-induced constipation in aged KM mice.

**Methods:**

Constipated mice were divided into groups receiving 10% lactulose (Y), hawthorn extract (S), probiotics (F), and the postbiotic of hawthorn-probiotic (FS). UPLC–MS metabolomics identified constituents of F, S, and FS. Network pharmacological analysis identified targets affected by FS. RT-qPCR assessed target expression in mouse colons, along with IL-6 and IL-17A levels. Molecular docking with AutoDock Tools1.5.6 evaluated interactions between FS components and targets. ex vivo colonic organ culture and RT-qPCR assessed target changes. Molecular dynamics analysis further scrutinized interactions. Targeted metabolomics measured short-chain fatty acid levels in mouse stool.

**Results:**

UPLC–MS metabolomics revealed distinct profiles for F, S, and FS, with FS showing decreased toxic substances and increased beneficial ones compared to S. Network pharmacology identified 20 cross-targets of FS in constipation. RT-qPCR showed decreased NR1I2 and SULT1A1 and increased GLP-2r in FS-treated mice. Inflammatory cytokines IL-6 and IL-17A were also reduced. ex vivo colonic organ culture and molecular docking identified effective combinations such as TNF-Baicalin and AQP3-Quinacridone. RMSD, RMSF, and RG analyses indicated favorable interactions between small molecules and targets. Targeted metabolomics revealed differing short-chain fatty acid contents in feces among groups.

**Conclusion:**

The postbiotic of hawthorn-probiotic alleviates constipation by regulating intestinal water and sodium metabolism, maintaining the intestinal barrier and gut flora, promoting epithelial cell proliferation, reducing inflammatory responses, and improving short-chain fatty acid metabolism.

## 1 Introduction

Chronic senile constipation is one of the common digestive system problems in the elderly (>65-70 years) (1, 2), and its incidence increases with the increase of age (3). This type of constipation can be caused by a variety of factors, including lifestyle factors, dietary habits, side effects of medications, neuromuscular dysfunction, and digestive disorders (4, 5). In addition to traditional drug treatment and dietary changes, some new treatment methods are gradually attracting attention, such as probiotics, prebiotics and post-biologics, which can relieve constipation by regulating intestinal flora and improving intestinal function (6–12).

Our previous research efforts have identified the potential of hawthorn-probiotic post-biotics in alleviating constipation (13). We found that postbiotic of hawthorn-probiotic exerts remarkable effects on constipation by regulating water and sodium metabolism, repairing intestinal barrier, relieving inflammation, and restoring microflora structure (13). Recently we make some progress on the postbiotic of hawthorn-probiotic metabolites and targets for constipation. We think these might offer a promising therapy for constipation.

## 2 Materials and methods

### 2.1 Materials

#### 2.1.1 Metabolite

**Table:** 

Pubchem CID	Metabolite name	Source	CAS number	Purity
13976	Quinacridone	Chengdu Must Bio-Technology Co., Ltd.	1047-16-1	98%
5282151	vitexin-2″-o-rhamnoside		64820-99-1	HPLC≥98%
9830628	L-Isoleucine		73-32-5	99%
64982	Baicalin		21967-41-9	HPLC≥98%
60198	Exemestane		107868-30-4	98%
5281643	Hyperoside		482-36-0	HPLC≥98%
5280343	Quercetin		117-39-5	HPLC≥98%
5280443	Apigenin		520-36-5	HPLC≥98%
8679	N-Phenyl-2-naphthylamine		135-88-6	98%
5280961	Genistein		446-72-0	97%
5281708	Daidzein		486-66-8	HPLC≥98%

#### 2.1.2 Short chain fatty acids standard

**Table:** 

Name	CAS number	Molecular formula	Molecular weight
Acetic acid	64-19-7	C_2_H_4_O_2_	60.05
Propionic acid	79-09-4	C_3_H_6_O_2_	74.08
Isobutyric acid	79-31-2	C_4_H_8_O_2_	88.11
Butyric Acid	107-92-6	C_4_H_8_O_2_	88.11
Valeric acid	109-52-4	C_5_H_10_O_2_	102.13
Isovaleric acid	503-74-2	C_5_H_10_O_2_	102.13
Hexanoic acid	142-62-1	C_6_H_12_O_2_	116.16

#### 2.1.3 Chemicals

**Table:** 

Name	Source	Cat. number
Methanol, LC–MS Grade	CNW	CAEQ-4-000306-4000
Acetonitrile, LC–MS Grade	CNW	CAEQ-4-000308-4000
Acetice Acid, LC–MS Grade	CNW	CAEQ-4-000319-0050
Ammonium Acetate, LC–MS Grade	CNW	CAEQ-4-000314-0050
Krebs-Ringer Buffer	Solarbio	G0430-500 ml

#### 2.1.4 Critical commercial assays

**Table:** 

Name	Source	Cat. number
RNAiso Plus	Vazyme	R401
SweScript RT I First Strand cDNA Synthesis Kit	Servicebio	G3330
2X Universal Blue SYBR Green qPCR Master Mix	Servicebio	G3326

#### 2.1.5 Software and Algorithms

**Table:** 

R version3.6.3	The R Foundation	N/A
SIMCA	Umetrics AB	N/A

### 2.2 Preparation and metabolic constituents of hawthorn-probiotic

#### 2.2.1 Preparation of hawthorn aqueous extract and postbiotic

According to the preparation process of the previous study (13), the sequence of *Lactobacillus paracasei* is shown in Supplementary Table S1. We prepared three groups of fermentation liquid required for the experiment [hawthorn group (S), probiotic group (F) and postbiotic of hawthorn-probiotic group (FS)].

#### 2.2.2 Metabolite extraction

The collected samples were thawed on ice, and metabolite were extracted with 80% methanol Buffer. Briefly, 100 μl of sample was extracted with 400 μl of precooled methanol. The extraction mixture was then stored in 30 min at −20°C. After centrifugation at 20,000*g* for 15 min, the supernatants were transferred into new tube to and vacuum dried. The samples were redissolved with 100 μl 80% methanol and stored at −80°C prior to the LC-MS analysis. In addition, pooled QC samples were also prepared by combining 10 μl of each extraction mixture (14).

#### 2.2.3 High performance liquid chromatography

All samples were acquired by the LC–MS system followed machine orders. Firstly, all chromatographic separations were performed using a Vanquish Flex UPLC system (Thermo Fisher Scientific, Bremen, Germany). An ACQUITY UPLC T3 column (100 mm*2.1 mm, 1.8 μm, Waters, Milford, USA) was used for the reversed phase separation. The column oven was maintained at 40°C. The flow rate was 0.3 mL/min and the mobile phase consisted of solvent A (water, 5 mM ammonium acetate and 5 mM acetic acid) and solvent B (Acetonitrile). Gradient elution conditions were set as follows: 0 ~ 0.8 min, 2% B; 0.8 ~ 2.8 min, 2 to 70% B; 2.8 ~ 5.3 min, 70 to 90% B; 5.3 ~ 5.9 min, 90 to 100% B; 5.9 ~ 7.5 min, 100% B; 7.5 ~ 7.6 min, 100 to 2% B; 7.6 ~ 10 min, 2%B.

#### 2.2.4 High resolution mass spectrometry

A high-resolution tandem mass spectrometer Q-Exactive (Thermo Scientific) was used to detect metabolites eluted from the column. The Q-Exactive was operated in both positive and negative ion modes. Precursor spectra (70–1,050 m/z) were collected at 70,000 resolution to hit an AGC target of 3e6. The maximum inject time was set to 100 ms. A top 3 configuration to acquire data was set in DDA mode. Fragment spectra were collected at 17,500 resolution to hit an AGC target of 1e5 with a maximum inject time of 50 ms. In order to evaluate the stability of the LC–MS during the whole acquisition, a quality control sample (Pool of all samples) was acquired after every 10 samples.

### 2.3 The analysis of the online pharmacology of post-biologics

#### 2.3.1 The collection and arrangement of the action target of the postbiotic of hawthorn-probiotic and constipation

TCMID, Batman and Herb databases were used to search for the corresponding target of the active ingredients of the postbiotic of hawthorn-probiotic.

No corresponding target ingredients were found for the time being, and Swisstargetprediction platform was used to predict the target. The results of these database searches would overlap. Excel software was used to remove duplicate targets and integrate, and the target name was imported into the Uniport database, and the type was set as “*Homo sapiens*” to obtain the corresponding gene name.

Disease targets by using “Constipation” as key word were searched and extracted by TTD, Herb and Omim databases.

#### 2.3.2 Analysis of Venn diagram and construction of target network of “TCM active ingredients-intersection target-disease”

Venny2.10 platform was used to analyze the target of drug action and the target of intestinal adhesion disease, and statistical intersection was formed and Wayne diagram was formed. The collected data were compiled and imported into Cytoscape3.91 software to construct the target network diagram of “TCM active ingredients—intersection targets—diseases.”

#### 2.3.3 PPI protein interaction network construction

The intersection targets were imported into the String platform and the type was defined as “*Homo sapiens*” to analyze and construct the PPI protein interaction network.

#### 2.3.4 Molecular docking

Acquisition and preprocessing of receptor proteins and compounds: The three-dimensional (3D) structures of the receptor proteins were obtained by jointly utilizing the UniProt^1^ and PDB^2^ databases. The protein structures were then processed using PyMOL version 2.3.0 to remove crystallographic water, irrelevant protein chains, and the original ligands. The 3D structures of the compounds were retrieved from the PubChem database,^3^ and the molecular structures were optimized using the MMFF94 force field with Open Babel3.1.1, resulting in the lowest energy conformation of the molecule. Detailed information about the compounds is provided in Table 1 at the end of the manuscript.

**Table 1:** The primers’ sequences of genes in this study.

Genes	Sequence of primers
*ABCB1B*	5′-AGTGTTAAAGGGGCGATGGG-3′
	5′-GACTCCTGTCCCGAGGTTTG-3′
*NR1L2*	5′-AGACCTGAGGAGAGCTGGAG-3′
	5′-TTGGCCTTGTCCCCACATAC-3′
*NR1L3*	5′-GTCCATGGGTTCCAGTACGAG-3′
	5′-TAACTCCGGGTCTGTCAGGG-3′
*SULT1A1 (ST1A1)*	5′-AGGTGATCTACGTTGCCCGAAATG-3′
	5′-GTACCACGACCCATAGGACACTTTC-3′
*IL-6*	5′-GGCGGATCGGATGTTGTGAT-3′
	5′-GGACCCCAGACAATCGGTTG-3′
*IL-17A*	5′-TGATGCTGTTGCTGCTGCTGAG-3′
	5′-CACATTCTGGAGGAAGTCCTTGGC-3′
*GLP-2r*	5′-TTCTGCCTCCTGCCGCTCTG-3′
	5′-AGTCCTTCAACCAGCAACCACAAG-3′
*AQP-3*	5′-CGCTGGTGTCTTCGTGTACC-3′
	5′-TGTGGGCCAGCTTCACATTC-3′
*ENAC-γ (SCNN1G)*	5′-TGAGTGACCTCCTGACTGACTTGG-3′
	5′-GAAATCTGGGTGGTGTGCCTTCC-3′
*TNF-α*	5′-GGAACACGTCGTGGGATAATG-3′
	5′-GGCAGACTTTGGATGCTTCTT-3′
*β-actin*	5′-CGTTGACATCCGTAAAGACC-3′
	5′-AACAGTCCGCCTAGAAGCAC-3′

Molecular Docking: Hydrogen atoms were added to both the receptor proteins and the compounds using AutoDock Tools1.5.6. The rotatable bonds of the compounds were identified, and the structures were saved in pdbqt format. The docking grid parameters were set using the Grid module. Receptor protein details and docking range parameters provided in Table 2. The docking protocol was set to semi-flexible docking, employing the Lamarckian genetic algorithm for docking with an exhaustiveness value of 25. Molecular docking was performed using AutoDock Vina 1.2.0 to obtain the binding free energy and docking result files.

**Table 2:** The top 10 metabolites of each protein by binding energy.

Protein name	Pubchem CID	Metabolite name	Binding energy
AQP3	13976	Quinacridone	−9.3
AQP3	5282151	Vitexin-2-O-rhamnoside	−9.3
AQP3	9830628	L-Isoleucine	−9.2
AQP3	64982	Baicalin	−9.1
AQP3	6198	EXEMESTANE	−8.5
AQP3	441793	Eurycomalactone	−8.4
AQP3	5281643	Hyperoside	−8.4
AQP3	76005853	6-[4-(3-Chlorophenyl)-1-Piperazinyl]-3-Cyclohexyl-2	−8.4
AQP3	6030	Uridine 5′-Monophosphate	−8.3
AQP3	13653606	Furanylfentanyl	−8.2
IL17A	64,982	Baicalin	−8.4
IL17A	13976	Quinacridone	−7.9
IL17A	441793	Eurycomalactone	−7.9
IL17A	60198	EXEMESTANE	−7.9
IL17A	76005853	6-[4-(3-Chlorophenyl)-1-Piperazinyl]-3-Cyclohexyl-2	−7.6
IL17A	9830628	L-Isoleucine	−7.5
IL17A	5280343	Quercetin	−7.3
IL17A	5282151	Vitexin-2-O-rhamnoside	−7.3
IL17A	13653606	Furanylfentanyl	−7
IL17A	5280443	Apigenin	−7
SCNN1G	13976	Quinacridone	−8.7
SCNN1G	5282151	Vitexin-2-O-rhamnoside	−8.3
SCNN1G	64982	Baicalin	−8.2
SCNN1G	13653606	Furanylfentanyl	−8.1
SCNN1G	5280448	Calycosin	−8
SCNN1G	9830628	L-Isoleucine	−8
SCNN1G	60198	EXEMESTANE	−7.9
SCNN1G	441793	Eurycomalactone	−7.7
SCNN1G	5281643	Hyperoside	−7.7
SCNN1G	76005853	6-[4-(3-Chlorophenyl)-1-Piperazinyl]-3-Cyclohexyl-2	−7.7
SULT1A1	8679	N-Phenyl-2-naphthylamine	−11.2
SULT1A1	5280443	Apigenin	−11.1
SULT1A1	5280343	Quercetin	−10.7
SULT1A1	15394713	(2S,4R)-4-(9H-Pyrido[3,4-b]indol-1-yl)-1,2,4-butanetriol	−10.4
SULT1A1	5280961	Genistein	−10.1
SULT1A1	6030	Uridine 5′-Monophosphate	−10
SULT1A1	10107340	N-Acetyl Retigabine	−9.9
SULT1A1	9830628	L-Isoleucine	−9.9
SULT1A1	5281708	Daidzein	−9.8
SULT1A1	575067	5-Hydroxy-4-methoxy-6-(2-phenylethyl)-5,6-dihydro-2H-pyran-2-one	−9.8
TNF	64982	Baicalin	−10.2
TNF	13653606	Furanylfentanyl	−9.7
TNF	6351946	L-prolyl-L-phenylalanine	−9.7
TNF	263426	(1xi,3S)-1,2,3,4-Tetrahydro-1-methyl-beta-carboline-1,3-dicarboxylic acid	−9.4
TNF	5280448	Calycosin	−9.4
TNF	78651	2-Amino-4-methylbenzophenone	−9.4
TNF	8679	N-Phenyl-2-naphthylamine	−9.4
TNF	5281708	Daidzein	−9.3
TNF	5280443	Apigenin	−9.2
TNF	73530	Tetrahydroharman-3-carboxylic acid	−9.2
GLP-2r	9830628	L-Isoleucine	−8.8
GLP-2r	5282151	Vitexin-2-O-rhamnoside	−8.7
GLP-2r	64982	Baicalin	−8.7
GLP-2r	441793	Eurycomalactone	−8.4
GLP-2r	13976	Quinacridone	−8.2
GLP-2r	263426	(1xi,3S)-1,2,3,4-Tetrahydro-1-methyl-beta-carboline-1,3-dicarboxylic acid	−8.2
GLP-2r	5281643	Hyperoside	−8.1
GLP-2r	60198	EXEMESTANE	−8
GLP-2r	580443	Apigeni	−7.9
GLP-2r	76005853	6-[4-(3-Chlorophenyl)-1-Piperazinyl]-3-Cyclohexyl-2	−7.9

#### 2.3.5 Molecular dynamics simulation

The Gromacs2020 software was used to simulate the molecular dynamics of the protein-compound complexes obtained by molecular docking. Amber14sb was selected as the protein force field, Gaff2 as the coordination force field, TIP3P water model was selected to add solvent to the protein ligand system and establish a water box with periodic boundary of 1.2 nm. Charges in the equilibrium system of sodium and chloride ions were added to restore the real experimental environment as far as possible. Prior to the formal kinetic simulation, the complex was minimized for 50,000 steps using a conjugate gradient algorithm, followed by a further equilibrium system of 100 ps using an isothermal (310 K) system (NVT) and an isobaric (1 standard atmosphere) system (NPT), and finally a molecular dynamics simulation of 100 Ns at normal temperature and pressure.

Analysis of molecular dynamics simulation results:

At present, the semi-flexible docking used for molecular docking cannot take into account the flexibility of protein structure. In order to further prove the degree and stability of binding between compounds and proteins, molecular dynamics simulations of 100 ns were performed for each group of docking results. We analyze the root mean square deviation (RMSD), root mean square fluctuation (RMSF), radius of rotation (Rg) and Gibbs free energy landscape in the molecular dynamics simulation trajectory of the complex.

### 2.4 Animals and groups

240-day-old, male KM mice obtained from the Guangzhou Regal Biotechnology Co., Ltd., SCXK [Yue] 2018–0182, SYXK [Yue] 2021–0059) were pair-housed in plastic cages in a temperature-controlled (25 ± 2°C) colony room under a 12/12-h light/dark cycle, with free access to food and water. The aged KM male mice were administrated with distilled water throughout the whole course and 6 mice were grouped as the normal controls without any other intervention(N). The rest mice were treated with 5 mg/kg loperamide (imodium, SOURCE: Xian Janssen Pharmaceutical Ltd. IDENTIFIER: LGJ0549.) for 1 week and randomly divided into model group (M), positive drug group (Y), hawthorn group (S), probiotic group (F) and postbiotic of hawthorn-probiotic group (FS). Mice in Y group were intragastrically treated with 10% lactulose (0.2 ml/day/per mouse), S group with 1 g/ml pure hawthorn solution (0.2 ml/day/per mouse), F group with *Lactobacillus paracasei* supernatant (0.2 ml/day/per mouse) and FS group with postbiotic of hawthorn-probiotic (0.2 ml/day/per mouse) for another week.

The inclusion criteria were normal development, appropriate age, and weight. The exclusion criteria were abnormal development, inappropriate age, or weight. Any single criterion not met resulted in exclusion. Grouping was conducted randomly according to cage order, and blind method was employed to select one individual for gavage. The experimenter assigned a number to the gavage material, and the individual performing the gavage administered it based on the assigned number. During the observation period, the condition of all KM mice was closely monitored. If any adverse reactions were detected, the gavage was immediately discontinued. However, throughout the entire experiment, the mice remained in good health and no abnormalities were observed.

These KM mice were sacrificed after anesthesia with pentobarbital sodium. The structure of colon, and fecal metabolite of mice were determined. All experimental protocols were approved by the Animal Center, Guangzhou University of Chinese Medicine.

### 2.5 RNA isolation and quantitative analysis

RNA was extracted from colon tissue using RNAiso Plus according to the instructions. Then, cDNA was obtained using the ImProm-II™ Reverse Transcription System (Promega) and RT-qPCR was carried out with custom designed oligonucleotides using the SweScript RT I First Strand cDNA Synthesis Kit and 2X Universal Blue SYBR Green qPCR Master Mix in a total volume of 20 μl: 95°C for 1 min and 40 cycles of denaturation (95°C for 15 s) and extension (60°C for 1 min). Experiments were performed in triplicates. Following amplification, dissociation curve analyses were performed to confirm the amplicon specificity for each PCR run. The relative level of gene expression in mouse colon tissue was normalized against mouse β-actin, respectively. Analysis of relative expression was performed using the 2(−ΔΔCT) method.

### 2.6 KM mouse ex vivo colonic organ culture

KM mouse (M group; with 5 mg/kg loperamide for 1 week; Constipation was diagnosed by insufficient fecal water content, long defecation time, small fecal volume and light weight) sacrificed after anesthesia with pentobarbital sodium. The colon were collected. The feces were removed with sterile syringes in a continuously ventilated (5% CO_2_ + 95% O_2_) Krebs-Ringer Buffer, and then cut colon into small segments 1 cm long. These colonal segments were then cut into sheets and placed in 24-well plates, respectively. Each well contained Krebs-Ringer Buffer continuously ventilated at 37°C and corresponding drug with a final concentration of 10 M and a volume of 1 ml. The culture was kept at 37°C, 5%CO_2_ + 95%O_2_, PH 7.4 for 5 h, and the tissue status was examined under the microscope every 15 min. Further experiments were carried out 5 h later.

### 2.7 Targeting metabolomics

The collected samples were thawed on ice, and metabolite were extracted with 80% methanol Buffer. Briefly, 50 mg of sample was extracted with 0.5 ml of precooled 80% methanol. The extraction mixture was then stored in 30 min at −20°C. After centrifugation at 20,000 g for 15 min, the supernatants were transferred into new tube to and vacuum dried. The samples were redissolved with 100 μl 80% methanol and stored at −80°C prior to the LC–MS analysis. In addition, pooled QC samples were also prepared by combining 10 μl of each extraction mixture.

Chromatographic mass spectrometry condition parameters are as follows: Chromatographic column: Waters ACQUITY UPLC BEH C18 (3.0 × 100 mm, 1.7 μm). Column temperature: 40°C. Sample size: 2 μl. Mobile phase: A: primary aqueous solution; B: Methanol: acetonitrile (volume ratio 1:1). The mass spectrum condition parameters are as follows: Sampling mode: MRM mode. See Materials section for standards. The standard curve is shown in Supplementary Table S2.

## 3 Results

### 3.1 The postbiotic of hawthorn-probiotic metabolites are quite different from the postbiotic of hawthorn and the postbiotic of hawthorn

**Figure 1:**
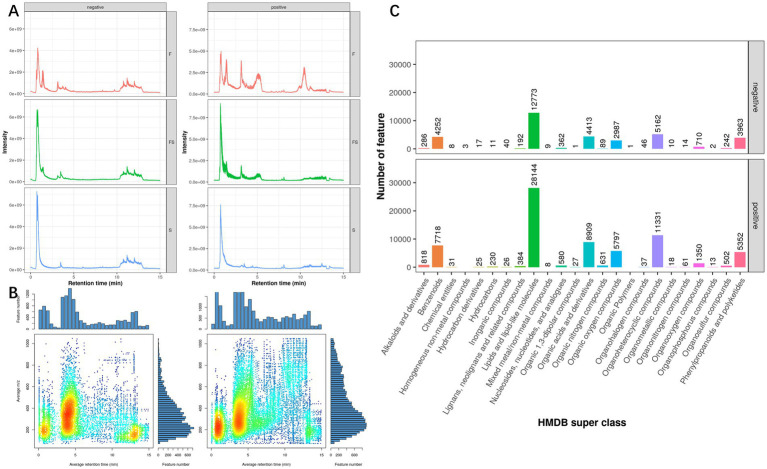
**(A)** TIC (Total Ion Chromotogram), a superposition of all metabolite strengths of each sample, with the retention time of the liquid chromatography as the horizontal coordinate and the ion strength as the vertical coordinate, reflecting the overall situation of the metabolite peak in the sample. **(B)** Mass-Charge Ratio and Retention time (mz-rt, mass-charge Ratio and Retention time). Each point is a metabolite peak, with the retention time of metabolites as the horizontal coordinate and the mass-charge ratio as the vertical coordinate, reflecting the overall hydrophilicity and molecular weight of all the metabolites in this project. The color of the dots represents the density of the region (the chromatographies are spectral series, red is dense, and blue is sparse). **(C)** Statistical map of metabolite species for first-order molecular weight identification. The figure extracts the classification information of the substance in which the substance is located and performs the number statistics. The horizontal coordinate is the classification and the vertical coordinate is the number of metabolic peaks.

The TIC (Total Ion Chromotogram) of the metabolites of Lactobacillus (F), postbiotic of hawthorn-probiotic(FS), hawthorn (S) is shown in Figure 1A. We found that after fermentation, the FS group was significantly different from the F and S groups in both anion and cationic tests. According to the Mz-rt figrure (Figure 1B), it can be found that the main detection period of metabolic ions in this test is 0–5 min, and the main detection m/z is 100–500. In this test, there were a total of 12,009 anion peaks and 20,611 cation peaks. The types of metabolic ion peaks are mainly lipids and lipid-like molecules, Organoheterocyclic compounds, Organic acids and derivatives, Benzenoids, Phenylpropanoids and polyketides, and Organic oxygen compounds.

**Figure 2:**
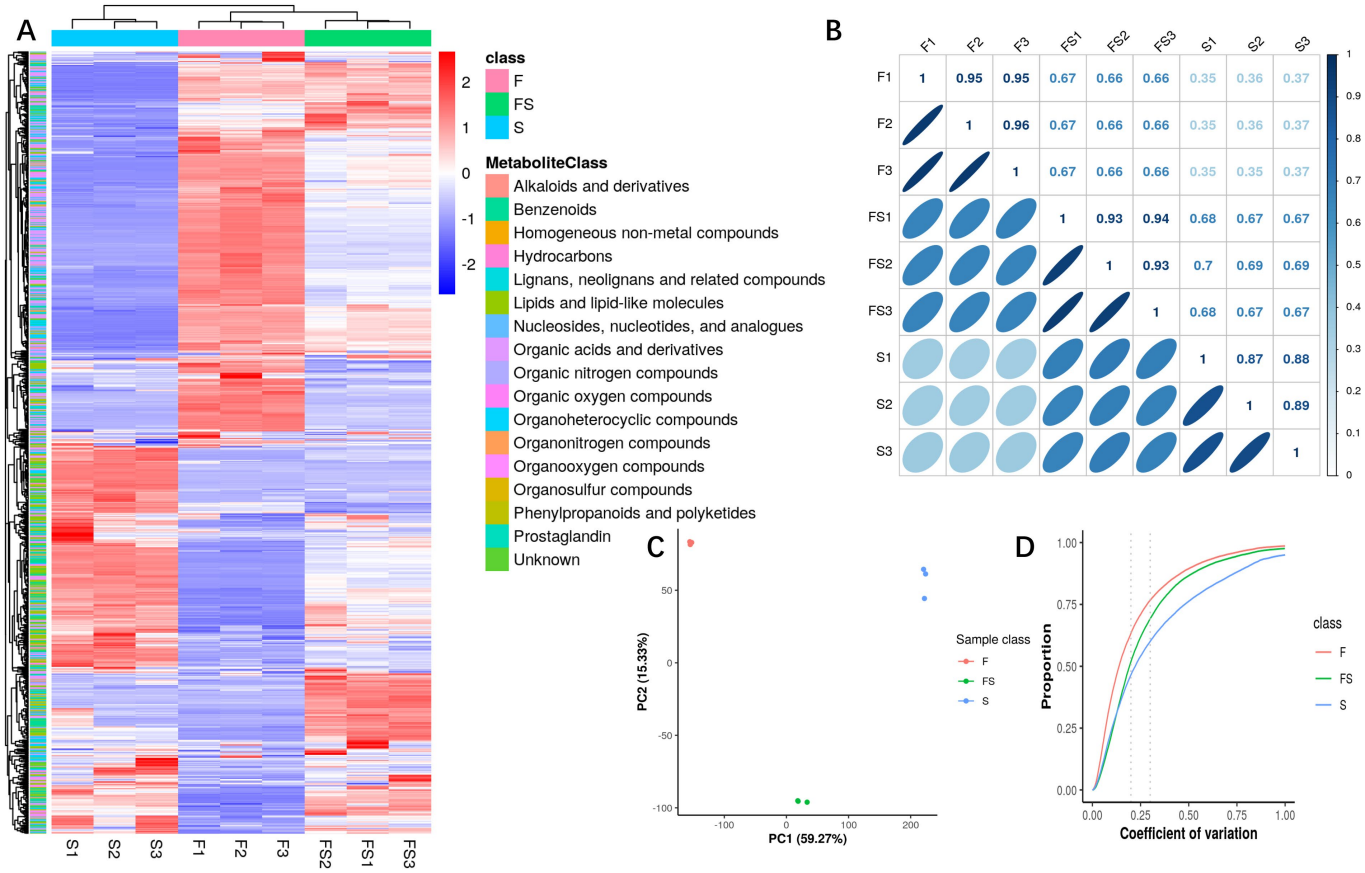
**(A)** Heatmap of metabolite intensity. Each column has a sample, each behavior has a displacement peak, and the color represents the intensity information (Log10 value). **(B)** Pairwise correlation heat map of samples. Pairwise sample correlation analysis was performed using all metabolic intensity of each sample. The upper right part of the figure shows the correlation coefficient, and the lower left part of the figure shows the degree of correlation fitting with an ellipse. **(C)** Principal component score chart. For PCA analysis of detected substances and identified metabolites, PCA score plot mainly represents the distribution trend of data points, each point in the graph represents a sample, and the similarities and differences between all samples are reflected in the separation trend or aggregation degree of samples in the score plot. The aggregation of points indicates that the observed variables have a high degree of similarity, and the dispersion of points indicates that the observed variables have obvious differences. **(D)** Biorepeat CV cumulative map. The difference coefficients of all replicates under each phenotype were calculated, and the cumulative statistics of each difference coefficient were carried out to show the repeated differences of different phenotypes. The horizontal coordinate of the CV cumulative curve represents the number of CV, and the vertical coordinate represents the proportion of metabolites smaller than the CV in the total number of metabolites.

After further analysis of primary metabolites (Figure 2A), we found that group FS integrated some components of group F and group S, and the part with effective increase in content was in the lower right corner of the way. As can be seen from Figures 2B–D, the differences within the test group were small, while the differences between the groups were large, and the biological repeatability was relatively good.

### 3.2 The postbiotic of hawthorn-probiotic is more abundant than the postbiotic of hawthorn and the postbiotic of hawthorn

**Figure 3:**
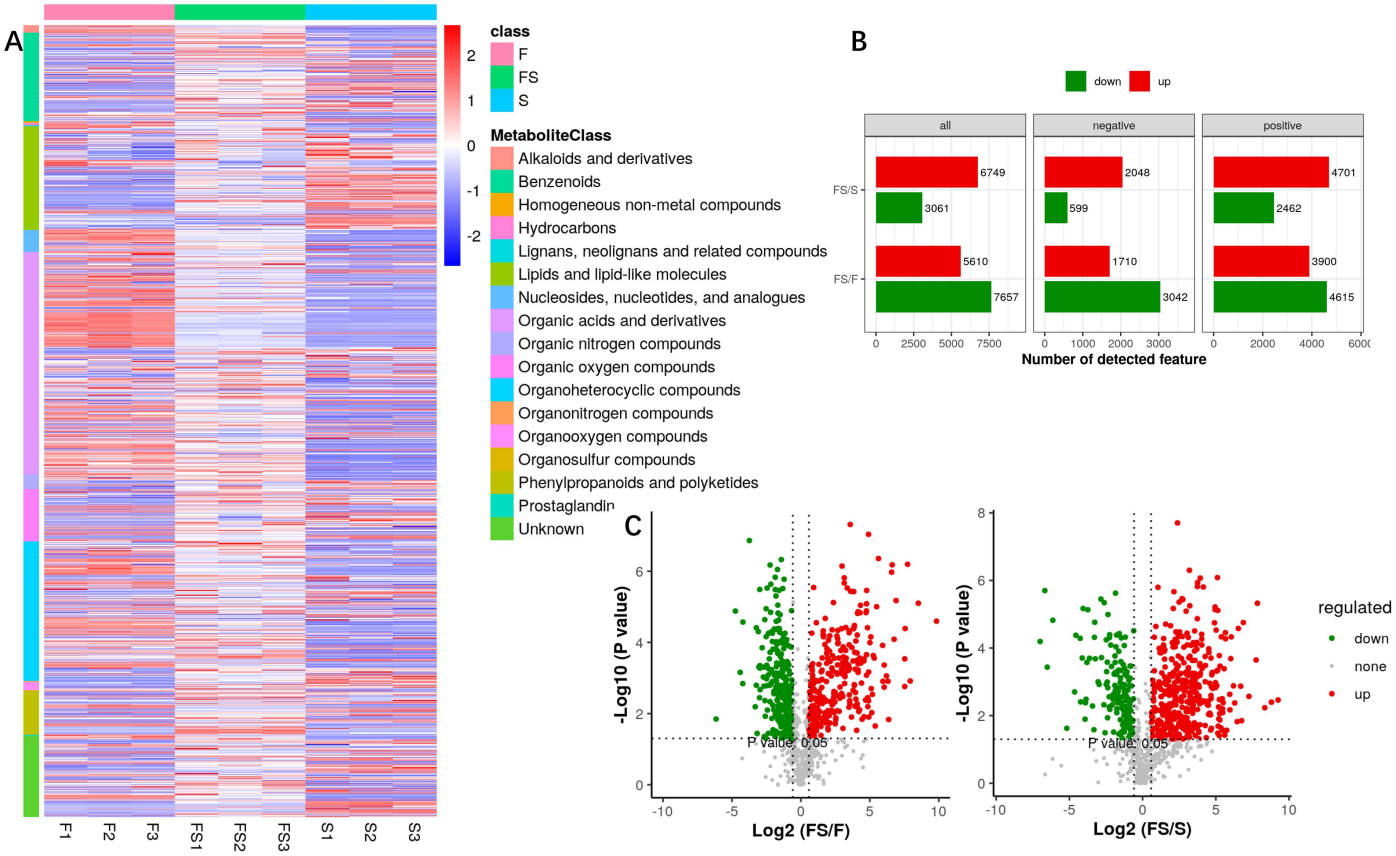
**(A)** Heatmap of the intensity of metabolites for secondary identification. Quantitative information was extracted from the secondary identified metabolites with high confidence, each of which was classified as a sample, each of which had a metabolic peak, and the color represented the intensity information (Log10 value). **(B)** Univariate analysis of fold-change and *p*-value obtained by T statistical test were used in this project to screen differentially expressed metabolites. Differential ions simultaneously satisfy: ratio > 1.5 or ratio < 1/1.5; *p* value < 0.05. **(C)** VolcanoPlot displays univariate statistical tests using volcanic maps. The horizontal coordinate is the ratio of the mean values of the two phenotypic strengths, and the vertical coordinate is the significance p-value (or corrected *p*-value) of the statistical test, and −log10 is taken. The greater the value, the more significant the difference.

**Figure 4:**
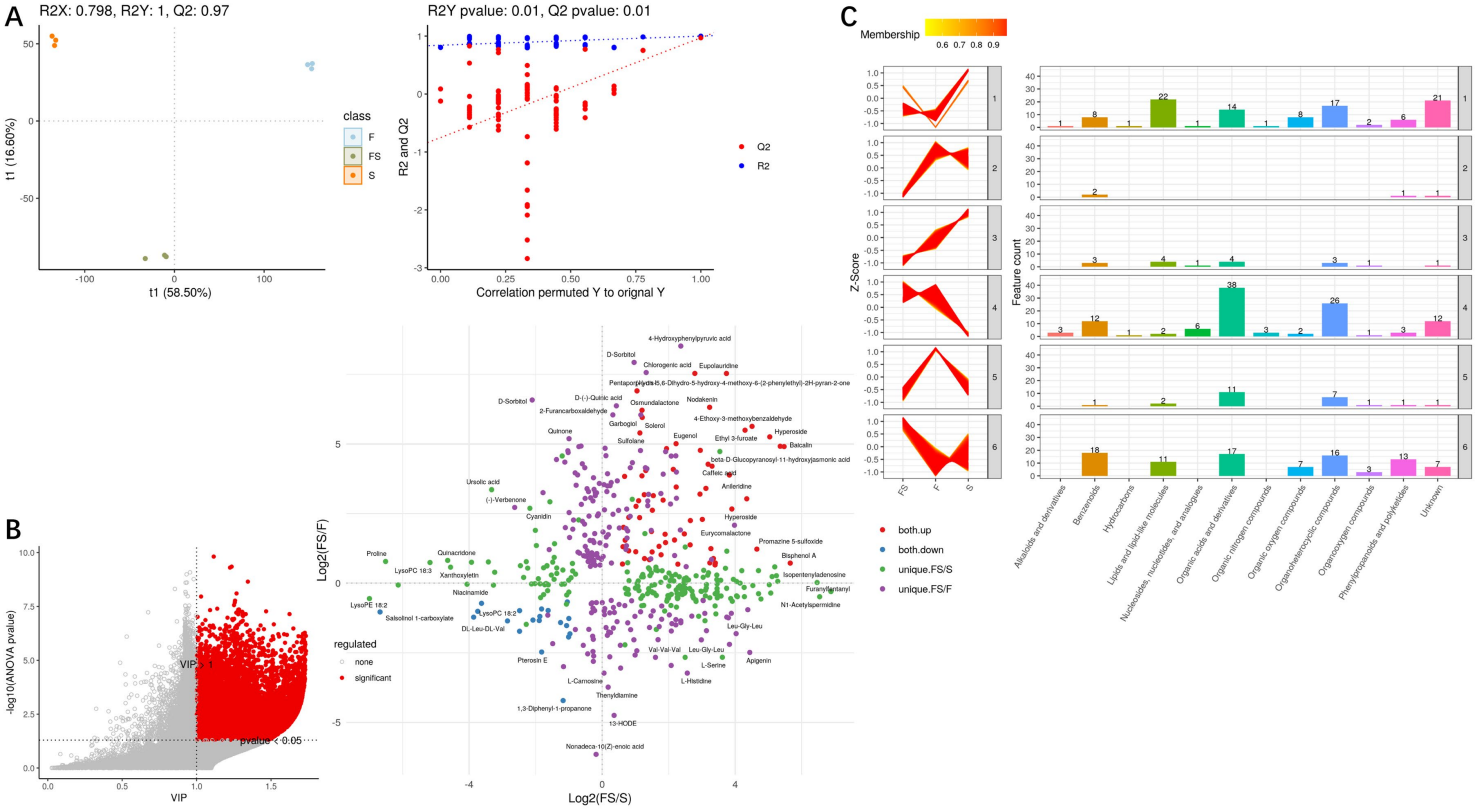
**(A)** PLS-DA score plots (left) are used to evaluate the relationships between samples, with the abscissa representing the first principal component and the ordinate representing the second principal component. Each dot represents a sample, and each color represents a phenotype; PLS-DA model check (right). The Permutation test tested whether the PLS-DA model was overfitted with a number of 100. The x-coordinate is the correlation of the Q2 and R2 calculated by the permutation to the original Q2 and R2 values, and the y-coordinate is the R2 and Q2 calculated by each permutation. Pvalue shows the significance of the replacement test, Q2’s pvalue< 0.05 indicates that the model is not overfitted. **(B)** For metabolites of global correlation in multiple comparison groups, metabolites with differential and secondary identification are extracted, each point of the correlation plot is a metabolite, and the horizontal and vertical coordinates are the ratio (logarithm) of each comparison group. **(C)** Multi-group secondary K clustering. The HMDB classification information was extracted from the metabolites identified by differential ions, and the metabolites were classified and counted for each expression category.

We analyzed the secondary metabolites (Figure 3A) and found that the three groups were still very different. FS/S mainly increased, while FS/F mainly decreased (Figure 3B). However, in general, the metabolite content of FS was higher than that of F and S (Figure 3C). As can be seen from Figure 4A, the difference within the test group is small, while the difference between groups is large, which is close to the real data.

With VIP > 1 and Pvalue <0.05, differential secondary metabolites were screened, and Figure 4B was obtained. Then, K-mean cluster analysis was performed on these differential secondary metabolites (Figure 4C), and it was found that Organic acids and derivatives, Organoheterocyclic compounds, lipids and lipid-like molecules, Benzenoids, Phenylpropanoids and polyketides are the main rising components of FS, but many components are unknown.

### 3.3 Network pharmacology and preliminary experiments have found common targets of constipation and postbiotic of hawthorn-probiotic

**Figure 5:**
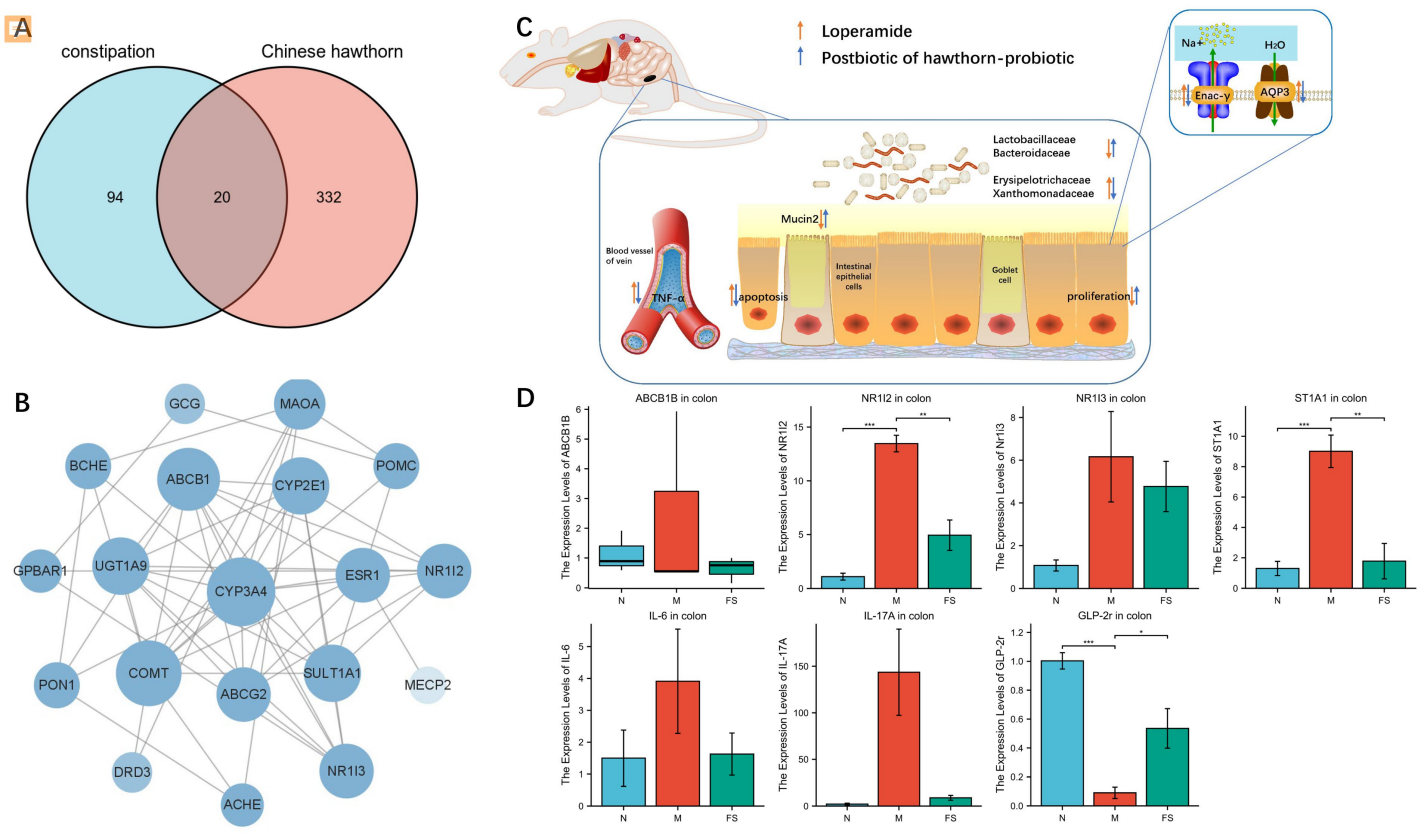
**(A)** Venn diagram, common target of constipation and fermentation fluid. **(B)** PPI maps of common targets. **(C)** Flow chart of the preliminary work. **(D)** Changes in target gene expression levels verified by colon RT-qPCR. NS; *p < 0.05; **p < 0.01; ***p < 0.001.

Then we went on target hunting. Through network pharmacology, we found 20 common targets for metabolic mixtures and constipation (Figure 5A). The PPI network between them is plotted (Figure 5B). In the previous study (Figure 5C), we also found and verified several other targets, including TNF-α, Mucin2, Enac-γ, and AQP3. Among them, TNF-α has attracted our attention, and after reviewing the literature, we also included IL-6 and IL-17A in the experiment. By conducting RT-qPCR on the colons of mice in normal group (N), model group (M), and postbiotic of hawthorn-probiotic group (FS), the primers are listed in Table 1. We found that the postbiotic of hawthorn-probiotic was capable of decreasing the expression of NR1I2 and ST1A1 (SULT1A1), and increased the expression of GLP-2r, a receptor for GCG. However, the expression of ABCB1B, NR1I3, IL-6, and IL-17A showed a decreased trend, with no statistical significance (Figure 5D).

### 3.4 Molecular docking and molecular dynamics analysis proved that the postbiotic of hawthorn-probiotic target metabolite was stable in binding to the target

**Figure 6:**
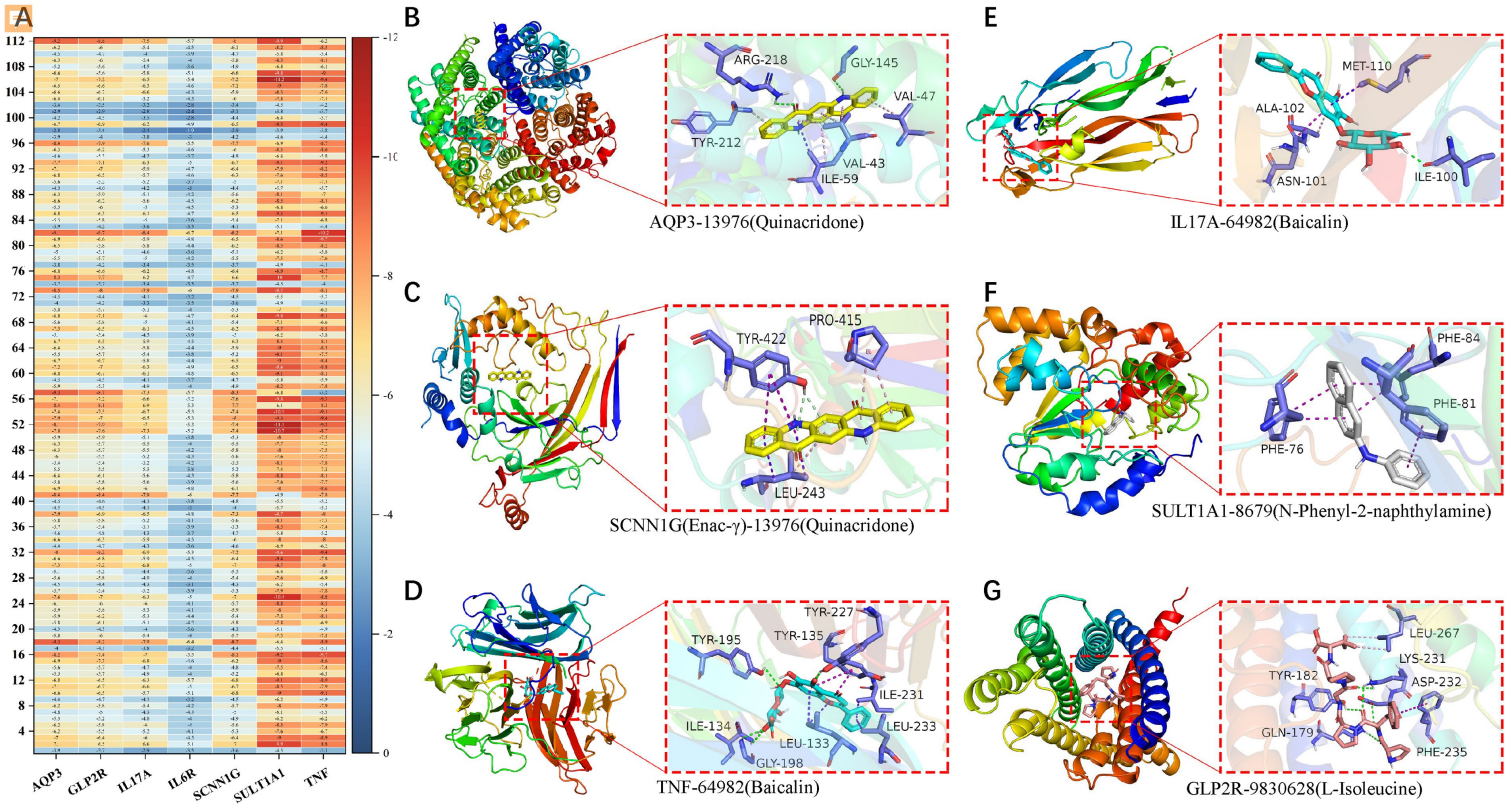
**(A)** Heat maps of binding energies of 112 secondary metabolites to 7 targets. Visualization of the combination with the highest binding energy of the **(B–G)** secondary metabolite and target protein.

**Figure 7:**
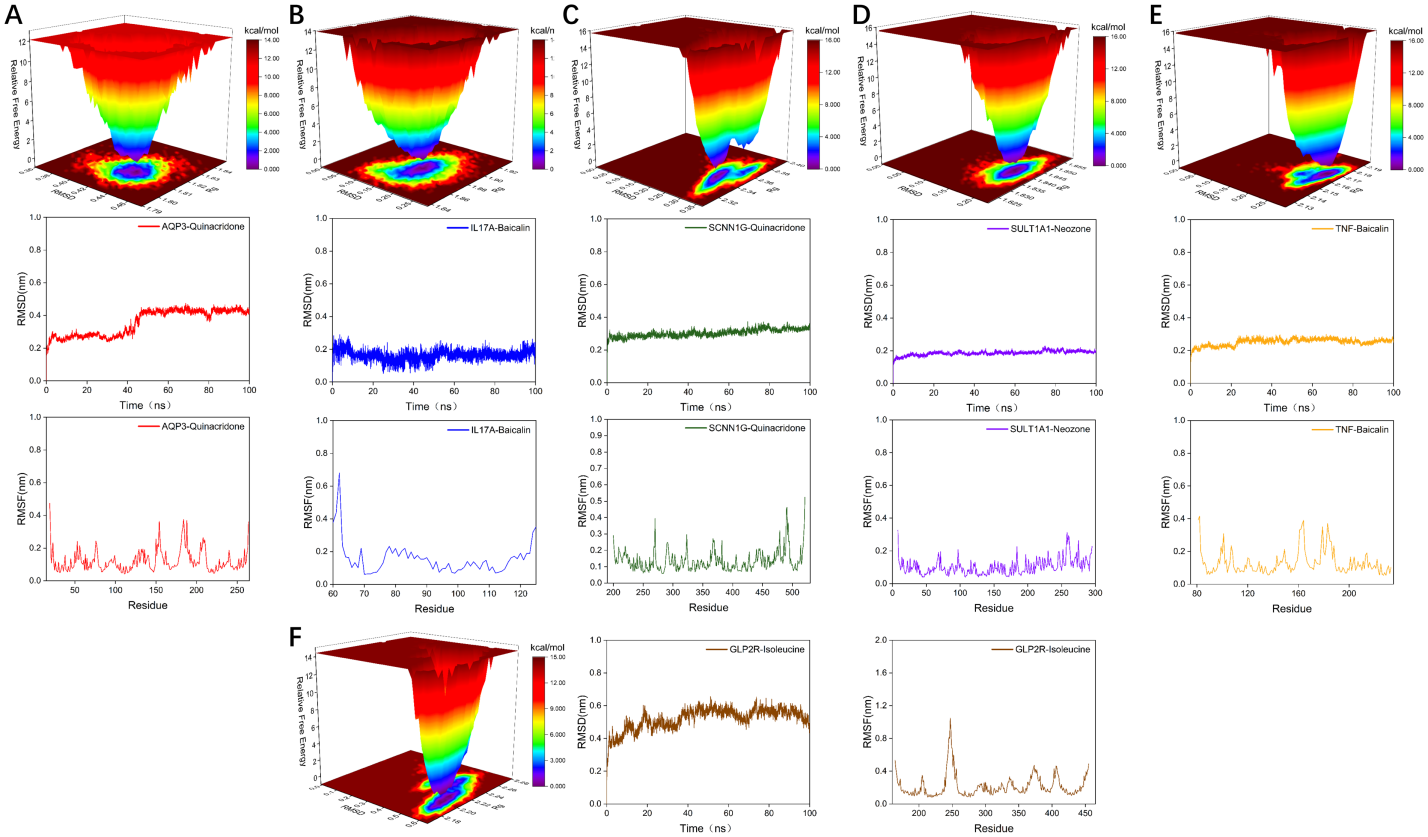
**(A–F)** Visualization of molecular dynamics analysis results of the combination with the highest binding energy of secondary metabolites and target proteins.

We found 131 significantly elevated secondary metabolites (see Supplementary material), and then searched for the postbiotic of hawthorn-probiotic that was significantly higher (VIP > 1.2, FC > 2or < 0.5, *p* < 0.01). We then used 112 metabolites for which we found a clear chemical structure to intermingle with previously screened targets (AQP3, Enac-γ, SULT1A1, TNF) and those we are still interested in (IL17A, IL6R). NR1I2 was found in the nucleus, and we did not focus on this target because we had no evidence that our metabolites could enter the nucleus. Subsequently, we found (Figure 6A) that these metabolites are generally combined with SULT1A1 and TNF. They bind well to parts of AQP3, GLP-2R, Enac-γ (SCNIG), and IL17A. But they do not combine well with IL6R. We used the index of binding energy to sort from large to small, and selected the top 10 compounds in each target for presentation (Table 2). Then, we combined the best groups and visualized them (Figures 6B–G). These are TNF-Baicalin, AQP3-Quinacridone, Enacγ-Quinacridone, IL17A-Baicalin, SULT1A1-N-Phenyl-2 naphthylamine, GLP2R-L-Isoleucine. We also performed molecular dynamics analysis on them (Figure 7). The results of RMSD, RMSF, and RG of the above small molecules and targets were all good.

### 3.5 Ex vivo colonic organ culture demonstrated that the target metabolite can be effective by loperamide to model the corresponding target of mouse colon

**Figure 8:**
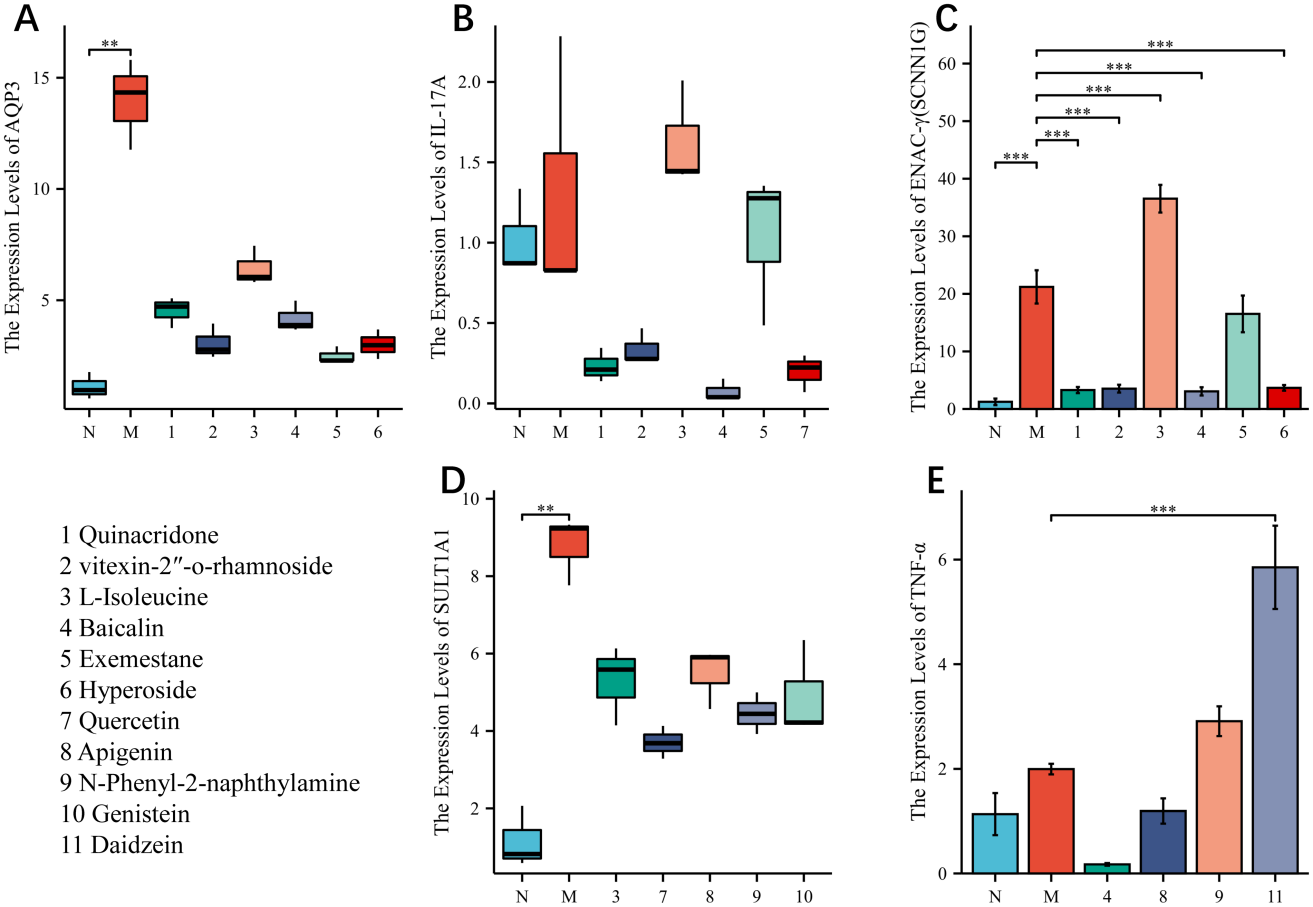
**(A–E)** *In vitro* experiments to verify the gene expression levels of the compound—target protein combinations with higher binding energy. NS; *p < 0.05; **p < 0.01; ***p < 0.001.

We tried our best to purchase the 11 compounds that were virtually screened (Quinacridone, vitexin-2 “-o-rhamnoside, L-Isoleucine, Baicalin, Exemestane, Hyperoside, Quercetin, Apigenin, N-Phenyl-2-naphthylamine, Genistein, Daidzein. See the Materials section for specific information), and the colons of loperamide modeled mice were removed for experiments. Finally, we found (Figure 8) that in general, the compound regulated the intestinal targets of the model mice in a favorable direction, especially Enac-γ. However, some compounds resulted in increased expression of the target, and there is a worsening trend. Daidzein, for example, increased TNF expression.

### 3.6 Targeted metabolomics found that postbiotic of hawthorn-probiotic regulated short chain fatty acids metabolism in the gut of loperamide modelled mice

**Figure 9:**
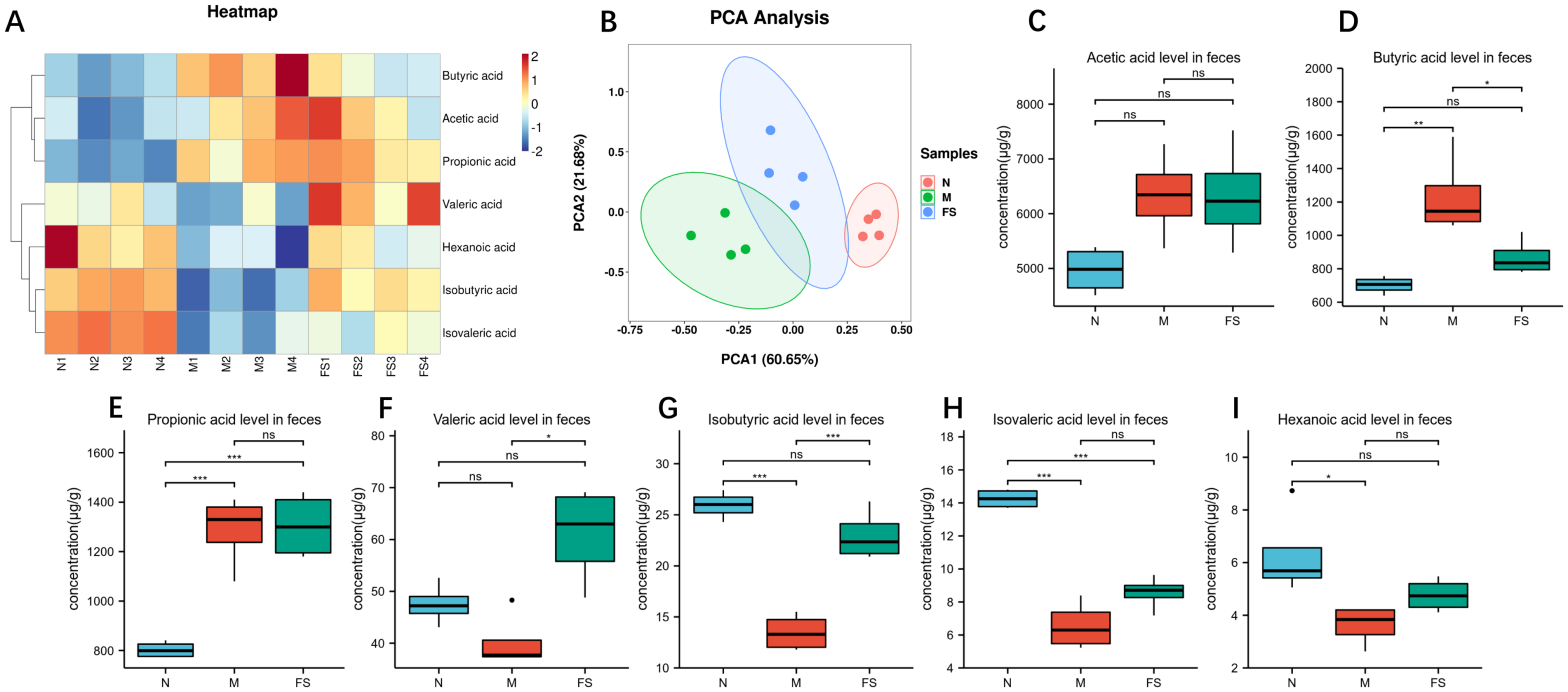
**(A)** Heat maps of various short chain fatty acids contents in feces of KM mice in groups N, M and FS. **(B)** PCA plots of the three groups. **(C–I)** Bar chart for the comparison of various short chain fatty acids contents in the three groups. NS; *p < 0.05; **p < 0.01; ***p < 0.001.

So we found that the postbiotic of hawthorn-probiotic regulated the gut microbiome structure of loperamide in mice and their ability to produce biofilms (13). So we conducted targeted metabolomic analysis on feces, and we found (Figure 9A) that the short chain fatty acids profiling of normal group (N), model group (M), postbiotic of hawthorn-probiotic (FS) was completely different. The three groups had good differentiation and intra-group repeatability (Figure 9B). By analyzing short chain fatty acids separately (Figures 9C–I), Butyric acid and Propionic acid contents rise significantly in the intestine of constipated mice, while Isovaleric acid and Hexanoic acid contents decline. The postbiotic of hawthorn-probiotic can reduce Butyric acid content to normal level, and it can also increase Valeric acid and Isobutyric acid content. However, Acetic acid and Propionic acid did not change significantly.

## 4 Discussion

With the exacerbation of population aging, constipation, a common gastrointestinal disease, is receiving increasing attention. As a novel approach, post-biotics are being studied in many diseases, including constipation. In our preliminary research (13), aged KM mice were modeled with loperamide, and we found that the postbiotic combination of hawthorn-probiotic was safe and effective. It alleviated intestinal water-sodium metabolism and chronic inflammation, promoted cell proliferation, and reduced apoptosis. By inhibiting harmful bacterial colonization, increasing the abundance of probiotics, and maintaining intestinal microbiota, it improved the gut microbiome. In this study, we observed an increase in the content of some secondary metabolites compared to hawthorn or probiotic alone. Through network pharmacology and mouse colonic tissue analysis, we identified new targets, particularly SULT1A1 and GLP-2r. Subsequently, molecular docking, molecular dynamics analysis, and *in vitro* experiments using colonic tissue from KM mice revealed stable binding between targets and secondary metabolites, especially TNF-Baicalin, AQP3-Quinacridone, Enacγ-Quinacridone, IL17A-Baicalin, SULT1A1-N-Phenyl-2 naphthylamine, and GLP2R-L-Isoleucine. Furthermore, short chain fatty acids metabolism analysis of feces from KM mice in this experiment indicated that the hawthorn-probiotic postbiotic improved short chain fatty acid profiling.

SULT1A1 is a sulfotransferase enzyme that participates in the metabolism and detoxification (15, 16) processes of various endogenous and exogenous compounds within the body (17). Its primary function involves conjugating electrophilic compounds with water-soluble sulfate substrates, thereby increasing their water solubility and facilitating their excretion from the body. In terms of research progress, SULT1A1 has been extensively studied and demonstrated significant roles in numerous fields (18, 19). For instance, in cancer research, the expression levels of SULT1A1 are closely associated with the occurrence and progression of tumors (20, 21), including colorectal cancer, by influencing tumor growth and metastasis through the regulation of endogenous hormone and carcinogen metabolism (17). Additionally, in drug metabolism and toxicology studies, SULT1A1 is also considered as one of the crucial enzymes involved in drug metabolism and detoxification, exerting significant effects on the metabolic pathways of many drugs and the formation of toxic metabolites (15). We found that the expression level of SULT1A1 in postbiotic of hawthorn-probiotic (FS) was lower than that in constipation model (M). This showed that the intestines of mice in FS group recovered and almost no toxins needed to be excreted.

GLP-2r, the receptor for the intestinal hormone glucagon-like peptide-2 (GLP-2), is widely expressed in intestinal tissues (22). GLP-2 is a gastrointestinal hormone secreted by enteroendocrine cells in the intestine (23), and its primary function is to promote proliferation and repair of intestinal mucosal cells, increase mucosal cell survival, while reducing intestinal motility and secretion (22, 24). These effects contribute to maintaining the integrity of the intestinal mucosa and promoting intestinal health. In terms of research progress, GLP-2r has been extensively studied and has shown potential roles in the treatment of various intestinal-related diseases. For example, GLP-2r agonists have been used in the treatment of inflammatory bowel diseases (such as Crohn’s disease and ulcerative colitis) (25) because they can promote intestinal mucosal repair and regeneration, improving patients’ symptoms and quality of life. Additionally, GLP-2r agonists are also being investigated for the treatment of other intestinal disorders such as intestinal fistulas and short bowel syndrome (22, 24). We found that the postbiotic of hawthorn-probiotic mice had elevated GLP-2r expression in colon. In previous studies, we observed by CCK8 and flow cytometry that postbiotic of hawthorn-probiotic promoted intestinal epithelial cell proliferation and reduced apoptosis. These suggest that the postbiotic of hawthorn-probiotic secondary metabolites bind to GLP-2r, promoting intestinal cell proliferation, reducing intestinal cell apoptosis, and restoring intestinal barrier function.

IL-17A is a cytokine belonging to the IL-17 family, primarily produced by T cells and other immune cells such as NK cells and lymphocytes (26), serving as a crucial inflammatory mediator (27, 28). It plays key roles in immune regulation, inflammatory responses (29) (particularly against bacterial and fungal infections (30)), and tissue repair (31). Inhibitors of IL-17A and its receptor have emerged as important targets for the treatment of autoimmune diseases (such as rheumatoid arthritis, psoriasis, etc.) and inflammatory bowel diseases (26). Relevant drugs have been developed and entered clinical trial phases. We found that the postbiotic of hawthorn-probiotic (FS) group had reduced IL-17A expression in colon tissue. So this suggests that the postbiotic of hawthorn-probiotic secondary metabolites are capable of reducing inflammation in the gut. Our previous studies found that postbiotic of hawthorn-probiotic could improve intestinal flora structure and short chain fatty acids metabolism in elderly constipated mice. IL-17A plays an important role in this.

In conclusion, our study once again proves that postbiotic of hawthorn-probiotic is beneficial to senile constipation, and the key secondary metabolites and targets are found and preliminarily verified. Our findings can be used as a reference for the treatment of constipation and other intestinal disorders.

## Data Availability

The original contributions presented in the study are included in the article/Supplementary material, further inquiries can be directed to the corresponding authors.

## Footnotes

^1^ https://www.uniprot.org/

^2^ https://www1.rcsb.org/

^3^ https://pubchem.ncbi.nlm.nih.gov/

## References

[1] Gallagher P O'Mahony D. Constipation in old age. Best Pract Res Clin Gastroenterol. (2009) 23:875–87. doi: 10.1016/j.bpg.2009.09.001, PMID: 19942165

[2] Barbara G Barbaro MR Marasco G Cremon C. Chronic constipation: from pathophysiology to management. Minerva Gastroenterol (Torino). (2023) 69:277–90. doi: 10.23736/S2724-5985.22.03335-6, PMID: 36727654

[3] Broad J Kung V Palmer A Elahi S Karami A Darreh-Shori T. Changes in neuromuscular structure and functions of human colon during ageing are region-dependent. Gut. (2019) 68:1210–23. doi: 10.1136/gutjnl-2018-316279, PMID: 30228216 PMC6594449

[4] Camilleri M Lee JS Viramontes B Bharucha AE Tangalos EG. Insights into the pathophysiology and mechanisms of constipation, irritable bowel syndrome, and diverticulosis in older people. J Am Geriatr Soc. (2000) 48:1142–50. doi: 10.1111/j.1532-5415.2000.tb04793.x, PMID: 10983917

[5] O'Mahony D O'Leary P Quigley EM. Aging and intestinal motility: a review of factors that affect intestinal motility in the aged. Drugs Aging. (2002) 19:515–27. doi: 10.2165/00002512-200219070-00005, PMID: 12182688

[6] Zhang J Chai X Zhao F Hou G Meng Q. Food applications and potential health benefits of hawthorn. Food Secur. (2022) 11:11. doi: 10.3390/foods11182861, PMID: 36140986 PMC9498108

[7] Ford AC Quigley EMM Lacy BE Lembo AJ Saito YA Schiller LR. Efficacy of prebiotics, probiotics, and synbiotics in irritable bowel syndrome and chronic idiopathic constipation: systematic review and meta-analysis. Am J Gastroenterol. (2014) 109:1547–61. doi: 10.1038/ajg.2014.202, PMID: 25070051

[8] Bazzocchi G Giovannini T Giussani C Brigidi P Turroni S. Effect of a new synbiotic supplement on symptoms, stool consistency, intestinal transit time and gut microbiota in patients with severe functional constipation: a pilot randomized double-blind, controlled trial. Tech Coloproctol. (2014) 18:945–53. doi: 10.1007/s10151-014-1201-5, PMID: 25091346

[9] Dan LW Luciana CL Amanda FB Raquel ST Glaucia MS Natalia PP. Effect of synbiotic in constipated adult women – a randomized, double-blind, placebo-controlled study of clinical response. Clin Nutr. (2013) 32:27–33. doi: 10.1016/j.clnu.2012.08.010, PMID: 22959620

[10] Ying JL Rosita J Abu SH Jin YC. Effects of Synbiotics among constipated adults in Serdang, Selangor, Malaysia—a randomised, double-blind, placebo-controlled trial. Nutrients. (2018) 10:10. doi: 10.3390/nu10070824, PMID: 29949873 PMC6073678

[11] Linetzky WD Alves PCC Logullo L Manzoni JT Almeida D Teixeira DSML. Microbiota benefits after inulin and partially hydrolized guar gum supplementation: a randomized clinical trial in constipated women. Nutr Hosp. (2012) 27:123–9. doi: 10.1590/S0212-16112012000100014, PMID: 22566311

[12] Ahmad K Mozhgan S. Role of Synbiotics in the treatment of childhood constipation: a double-blind randomized placebo controlled trial. Iran J Pediatr. (2010) 20:387–92. PMID: 23056736 PMC3446081

[13] Wei Y Huang N Ye X Liu M Wei M Huang Y. The postbiotic of hawthorn-probiotic ameliorating constipation caused by loperamide in elderly mice by regulating intestinal microecology. Front Nutr. (2023) 10:1103463. doi: 10.3389/fnut.2023.1103463, PMID: 37006920 PMC10061020

[14] Dunn WB Broadhurst D Begley P Zelena E Francis-McIntyre S Anderson N. Procedures for large-scale metabolic profiling of serum and plasma using gas chromatography and liquid chromatography coupled to mass spectrometry. Nat Protoc. (2011) 6:1060–83. doi: 10.1038/nprot.2011.335, PMID: 21720319

[15] Cook I Wang T Leyh TS. Isoform-specific therapeutic control of sulfonation in humans. Biochem Pharmacol. (2019) 159:25–31. doi: 10.1016/j.bcp.2018.11.010, PMID: 30423313 PMC6625639

[16] Tyapochkin E Kumar VP Cook PF Chen G. Reaction product affinity regulates activation of human sulfotransferase 1A1 PAP sulfation. Arch Biochem Biophys. (2011) 506:137–41. doi: 10.1016/j.abb.2010.11.018, PMID: 21111704 PMC3049928

[17] Suzuki H Morris JS Li Y Doll MA Hein DW Liu J. Interaction of the cytochrome P4501A2, SULT1A1 and NAT gene polymorphisms with smoking and dietary mutagen intake in modification of the risk of pancreatic cancer. Carcinogenesis. (2008) 29:1184–91. doi: 10.1093/carcin/bgn085, PMID: 18499698 PMC2443278

[18] Pietrauszka K Bergler-Czop B. Sulfotransferase SULT1A1 activity in hair follicle, a prognostic marker of response to the minoxidil treatment in patients with androgenetic alopecia: a review. Postepy Dermatol Alergol. (2022) 39:472–8. doi: 10.5114/ada.2020.99947, PMID: 35950120 PMC9326921

[19] Paul S Gangwar A Patir H Bhargava K Ahmad Y. Reverse translating SULT1A1, a potential biomarker in roentgenographically tested rat model of rapid HAPE induction. Life Sci. (2019) 229:132–8. doi: 10.1016/j.lfs.2019.05.035, PMID: 31100325

[20] Shi L Shen W Davis MI Kong K Vu P Saha SK. SULT1A1-dependent sulfonation of alkylators is a lineage-dependent vulnerability of liver cancers. Nat Can. (2023) 4:365–81. doi: 10.1038/s43018-023-00523-0, PMID: 36914816 PMC11090616

[21] Santos SS Koifman RJ Ferreira RM Diniz LF Brennan P Boffetta P. SULT1A1 genetic polymorphisms and the association between smoking and oral cancer in a case-control study in Brazil. Front Oncol. (2012) 2:183. doi: 10.3389/fonc.2012.00183, PMID: 23264952 PMC3524504

[22] Cheng W Wang K Zhao Z Mao Q Wang G Li Q. Exosomes-mediated transfer of miR-125a/b in cell-to-cell communication: a novel mechanism of genetic exchange in the intestinal microenvironment. Theranostics. (2020) 10:7561–80. doi: 10.7150/thno.41802, PMID: 32685005 PMC7359098

[23] Lee SJ Lee J Li KK Holland D Maughan H Guttman DS. Disruption of the murine Glp2r impairs Paneth cell function and increases susceptibility to small bowel enteritis. Endocrinology. (2012) 153:1141–51. doi: 10.1210/en.2011-1954, PMID: 22253424 PMC3606134

[24] Austin K Imam NA Pintar JE Brubaker PL. IGF binding protein-4 is required for the growth effects of glucagon-like peptide-2 in murine intestine. Endocrinology. (2015) 156:429–36. doi: 10.1210/en.2014-1829, PMID: 25514089 PMC4298331

[25] Shinzaki S Sato T Fukui H. Antidiabetic drugs for IBD: a long but promising road ahead for drug repositioning to target intestinal inflammation. J Gastroenterol. (2023) 58:598–9. doi: 10.1007/s00535-023-01983-y, PMID: 36961556

[26] Akitsu A Iwakura Y. Interleukin-17-producing gammadelta T (gammadelta17) cells in inflammatory diseases. Immunology. (2018) 155:418–26. doi: 10.1111/imm.12993, PMID: 30133701 PMC6231014

[27] Chung SH Ye XQ Iwakura Y. Interleukin-17 family members in health and disease. Int Immunol. (2021) 33:723–9. doi: 10.1093/intimm/dxab075, PMID: 34611705 PMC8633656

[28] McGeachy MJ Cua DJ Gaffen SL. The IL-17 family of cytokines in health and disease. Immunity. (2019) 50:892–906. doi: 10.1016/j.immuni.2019.03.021, PMID: 30995505 PMC6474359

[29] Grizotte-Lake M Zhong G Duncan K Kirkwood J Iyer N Smolenski I. Commensals suppress intestinal epithelial cell retinoic acid synthesis to regulate Interleukin-22 activity and prevent microbial Dysbiosis. Immunity. (2018) 49:1103–1115.e6. doi: 10.1016/j.immuni.2018.11.018, PMID: 30566883 PMC6319961

[30] Conti HR Bruno VM Childs EE Daugherty S Hunter JP Mengesha BG. IL-17 receptor signaling in Oral epithelial cells is critical for protection against oropharyngeal candidiasis. Cell Host Microbe. (2016) 20:606–17. doi: 10.1016/j.chom.2016.10.001, PMID: 27923704 PMC5147498

[31] Göbel K Pankratz S Asaridou CM Herrmann AM Bittner S Merker M. Blood coagulation factor XII drives adaptive immunity during neuroinflammation via CD87-mediated modulation of dendritic cells. Nat Commun. (2016) 7:11626. doi: 10.1038/ncomms11626, PMID: 27188843 PMC4873982

