# Supplementary material for: Multi-omics revealed that the postbiotic of hawthorn-probiotic alleviated constipation caused by loperamide in elderly mice
Source: Front Nutr. 2025 Feb 18;12:1498004. doi: 10.3389/fnut.2025.1498004 (PMC11895004; doi:10.3389/fnut.2025.1498004)
Supplement: Supplementary file 1 [file Table_1.pdf]

Supplemental Materials

1.Supplemental tables

Table S1: 16s rRNA sequence of *Lactobacillus paracasei*.

Results of strain identification	Sequence
Lactobacillus paracasei subsp. tolerans	GGGGGCATGGCGGCGTGCTATACATGCAAGTCGAACGAGTTCT<br>CGTTGATGATCGGTGCTTGCACCGAGATTCAACATGGAACGAG<br>TGGCGGACGGGTGAGTAACACGTGGGTAACCTGCCCTTAAGTG<br>GGGGATAACATTTGGAAACAGATGCTAATACCGCATAGATCCA<br>AGAACCGCATGGTTCTTGGCTGAAAGATGGCGTAAGCTATCGC<br>TTTTGGATGGACCCGCGGCGTATTAGCTAGTTGGTGAGGTAAT<br>GGCTCACCAAGGCGATGATACGTAGCCGAACTGAGAGGTTGAT<br>CGGCCACATTGGGACTGAGACACGGCCCAAACTCCTACGGGAG<br>GCAGCAGTAGGGAATCTTCCACAATGGACGCAAGTCTGATGGA<br>GCAACGCCGCGTGAGTGAAGAAGGCTTTCGGGTCGTAAAACTC<br>TGTTGTTGGAGAAGAATGGTCGGCAGAGTAACTGTTGTCGGCG<br>TGACGGTATCCAACCAGAAAGCCACGGCTAACTACGTGCCAGC<br>AGCCGCGGTAATACGTAGGTGGCAAGCGTTATCCGGATTTATT<br>GGGCGTAAAGCGAGCGCAGGCGGTTTTTTAAGTCTGATGTGAA<br>AGCCCTCGGCTTAACCGAGGAAGCGCATCGGAAACTGGGAAAC<br>TTGAGTGCAGAAGAGGACAGTGGAACTCCATGTGTAGCGGTGA<br>AATGCGTAGATATATGGAAGAACACCAGTGGCGAAGGCGGCT<br>GTCTGGTCTGTAACTGACGCTGAGGCTCGAAAGCATGGGTAGC<br>GAACAGGATTAGATACCCTGGTAGTCCATGCCGTAAACGATGA<br>ATGCTAGGTGTTGGAGGGTTTCCGCCCTTCAGTGCCGCAGCTAA<br>CGCATTAAGCATTCCGCCTGGGGAGTACGACCGCAAGGTTGAA<br>CTCAAAGGAATTGACGGGGGCCCGCACAAGCGGTGGAGCATGT<br>GGTTTAATTCGAGCACCGCGAGACCCTACCAGTCTTGACATCTT<br>TTGATCACCTTGAGAGATCAGGTTTCCCCTTCCGGGGCAAATGA<br>CAGGTGGTGCATGTTGTTCGTCAGCCTCGTGTCGTGAGATGTTG<br>GGTAGTTCCCGCAACGAAGCGCAACCTCATATAG

Table S2: Target amino acid metabolomics standard curve:

NO.	Name	Retention time （min）	Linear range （μg/g）	Linear equation	R^2	content （μg/ g）
1	Acetic acid	4.4	5.00~100	Y = 5.83062e+006+547799*X	0.9975	0.04
2	Propionic acid	6.1	5.00~50.0	Y = 3.07462e+006+1.7952e+006*X	0.9964	0.04
3	Isobutyric acid	8.1	1.00~10.0	Y = 640570+3.747e+006*X	0.9986	0.04
4	Butyric acid	8.5	5.00~50.0	Y = 2.20867e+006+2.0584e+006*X	0.9985	0.1
5	Isovaleric acid	10.8	1.00~10.0	Y = 225653+2.78265e+006*X	0.9974	0.02
6	Valeric acid	11.4	1.00~10.0	Y = 378198+2.35145e+006*X	0.9987	0.02
7	Caproic acid	14.2	0.100~1.00	Y = 121397+3.49737e+006*X	0.9992	0.02
